# Age-Dependent Decrease in Adropin is Associated with Reduced Levels of Endothelial Nitric Oxide Synthase and Increased Oxidative Stress in the Rat Brain

**DOI:** 10.14336/AD.2017.0523

**Published:** 2018-04-01

**Authors:** Changjun Yang, Kelly M. DeMars, Eduardo Candelario-Jalil

**Affiliations:** Department of Neuroscience, McKnight Brain Institute, University of Florida, Gainesville, FL, USA; Department of Neuroscience, McKnight Brain Institute, University of Florida, Gainesville, FL, USA; Department of Neuroscience, McKnight Brain Institute, University of Florida, Gainesville, FL, USA

**Keywords:** adropin, aging, endothelial nitric oxide synthase, gp91^phox^, 4-hydroxynonenal

## Abstract

Adropin is a peptide highly expressed in the brain. Emerging evidence indicates that low plasma levels of adropin are closely associated with aging and endothelial dysfunction. We hypothesized that aging reduces adropin levels in the brain, which correlates with reduced endothelial nitric oxide synthase (eNOS) and increased oxidative stress associated with age-related endothelial dysfunction. Cortical brain tissue and plasma were collected from young (10-12 weeks old) and aged (18-20 months old) male Sprague-Dawley naïve rats. Using RT-qPCR, we quantified the mRNA levels of the *energy homeostasis associated (Enho)* gene encoding for adropin. Western blotting was utilized to measure adropin and markers of endothelial dysfunction and oxidative stress in the brain tissue. Levels of adropin in plasma were measured using an ELISA kit. Compared to young rats, both *Enho* mRNA and protein levels were dramatically reduced in the aged rat brain, which was accompanied by a significant reduction in plasma adropin levels in aged compared to young rats. Additionally, total and phosphorylated levels of endothelial nitric oxide synthase (eNOS) were significantly decreased in aged rat brains and were associated with dramatically increased gp91^phox^-containing NADPH oxidase (a major source of free radicals) and 4-hydroxynonenal (4-HNE), a lipid peroxidation marker. Brain levels of Akt and caveolin-1 were significantly reduced in aged rats compared with young animals. Collectively, these findings indicate that adropin levels negatively correlate with markers of endothelial dysfunction and oxidative injury, which raises the possibility that loss of brain adropin might play a role in the pathogenesis and development of aging-associated cerebrovascular dysfunction.

Adropin is a recently identified and highly conserved polypeptide, encoded by the *energy homeostasis associated* gene (*Enho*), consisting of a secretory signal peptide by N-terminal amino acid residues 1-33 and a putative secreted domain of 34-76 amino acids, which is highly expressed in the brain [1, 2].

Adropin exerts beneficial effects on regulating endothelial function through increasing angiogenesis, blood flow, and capillary density in a mouse model of hind limb ischemia [3]. Further findings revealed that adropin is expressed in vascular endothelial cells, and treatment with adropin reduced endothelial permeability through activation of endothelial nitric oxide synthase (eNOS)/NO signaling pathways [3]. We have shown that adropin is protective in brain endothelial cells exposed to hypoxia and low glucose *in vitro* by attenuating increased endothelial permeability in a concentration-dependent manner, and found that the gene encoding adropin, *Enho*, was dramatically downregulated in rat brain microvascular endothelial cells exposed to ischemia-like conditions [4]. There is emerging evidence that low levels of plasma adropin are closely associated with endothelial dysfunction in patients with diabetes or cardiovascular diseases [5-8]. This is in line with our recent work showing significantly decreased adropin levels in the ischemic mouse brain after stroke, and dose-dependent reduction of stroke-induced infarct volume with synthetic adropin treatment. Further findings revealed that adropin-mediated neuroprotection was through eNOS activation and reduced oxidative damage [9]. In humans, age was negatively correlated with plasma adropin levels, where individuals aged 30 years and younger showed much higher plasma adropin levels than those subjects between 40-50 or 50 years and older [10]. However, there is no work addressing the relationship between the brain adropin levels in aged subjects and aging-associated cerebrovascular dysfunction, which may potentially contribute to incidences of stroke.

In this study, we hypothesized that aging will affect adropin levels in the rat brain and plasma, which correlates with reduced endothelial nitric oxide synthase (eNOS) and increased oxidative stress associated with age-related endothelial dysfunction. Utilizing the same strains of young and aged naïve rats, we found for the first time that *Enho* mRNA and protein levels were significantly downregulated in the aged rat brain, and this was associated with decreased plasma adropin. Further findings revealed that the reduced brain and plasma adropin levels in aged rats were associated with decreased total and phosphorylated eNOS, as well as increased oxidative stress markers in brain. Collectively, these data suggest that adropin may play an important role in aging-associated cerebrovascular diseases.

## MATERIALS AND METHODS

### Animals, *a priori* sample size calculation and power analysis

All experimental procedures were in accordance with the National Institutes of Health guidelines for rodents and protocols were approved by the University of Florida Institutional Animal Care and Use Committee. In this study, five young (10-12 weeks old) and five old (18-20 months old) male Sprague Dawley rats (Hilltop Laboratories, Scottdale, PA) were used. Based on our pilot studies, we performed an *a priori* sample size calculation using the G*Power v.3.1.9.2 software [11]. To calculate Cohen effect size (*d*), we compared two independent groups in a two-tailed unpaired *t*-test using α=0.05, and β (type II error) of 0.1 with a power of 90%. We utilized means and standard deviations from a pilot study measuring brain adropin levels by immunoblotting in the rat brain. We calculated a sample size of *n*=5 with an effect size of *d*=2.09. All animals were acclimated to our animal facility for at least 7 days before sacrifice. All rats were housed in a controlled environment maintained under a 12-h light/dark cycle, and they had free access to food and water. At the time of sacrifice, rats were deeply anesthetized with pentobarbital (150 mg/kg; i.p.) and peripheral venous blood was drawn from the inferior *vena cava* and blood samples were immediately transferred into EDTA-coated tubes and centrifuged for 10 min at 2,000×*g* and plasma samples were stored at -80 °C for plasma adropin measurement using a commercial ELISA kit. Rats were perfused intracardially with ice-cold saline and cerebral cortex was collected for RNA and protein isolation.

### RNA isolation and preparation of brain lysates

Cerebral cortical tissue was homogenized in radioimmunoprecipitation lysis buffer consisting of 50 mM Tris-HCl (pH 7.4), 150 mM NaCl, 5 mM EDTA, 1% NP-40, 1% sodium deoxycholate and 1% SDS plus Protease and Phosphatase Inhibitor Cocktails (Cat. Nos. 78430 and 78428, respectively; Thermo Fisher Scientific, Rockford, IL). Then, half of this tissue lysate was mixed with an equal volume of denaturing solution consisting of 4 M guanidinium thiocyanate, 25 mM sodium citrate (pH 7.0), 0.5% (wt/vol) *N*-laurosylsarcosine (Sarkosyl) and 0.1 M β-mercaptoethanol for RNA isolation and quantification as previously reported in details [12]. The rest of the tissue lysate was used for protein extraction as described in our previous report [13].

### Quantitative Real-Time PCR

RNA was transcribed into cDNA using iScript Reverse Transcription Supermix (Cat. No. 170-8841; Bio-Rad, Hercules, CA) according to the manufacturer’s instructions. Quantitative real-time PCR was performed with 20?ng of cDNA in a total volume of 10?μL using SYBR Green Supermix (Cat. No. 172-5272; Bio-Rad) according to the manufacturer’s protocol. The primer sequences used for amplification of rat *Enho* gene were: forward, 5′-GCTCAACTCAGGCTCAGGAC-3′; and reverse, 5′-CGACTTTCCAAGGAGGCTGT-3′ and were normalized to the housekeeping gene *Hprt1*: forward, 5′- GGTGAAAAGGACCTCTCGAAG-3′; and reverse, 5′- GCTTTTCCACTTTCGCTGATG-3′. PCR reactions were run in triplicate and cycle threshold values were normalized to *Hprt1* expression for each sample.

### Immunoblotting

Fifty micrograms of total protein were incubated in non-reducing Laemmli’s sample buffer for 5 min at 100 °C to measure gp91^phox^ or at 60 °C for 3 min to measure 4-hydroxynonenal (4-HNE) as previously reported [14]. To measure adropin, phosphorylated eNOS at Ser1176 (peNOS^Ser1176^), total eNOS, Akt, caveolin-1, and β-actin, samples were incubated in reducing Laemmli’s sample buffer for 5 min at 100 °C prior to loading samples. All samples were separated on 4-20% SDS-polyacrylamide gels and then transferred onto nitrocellulose membranes. Membranes were then blocked for 1 h at room temperature with 5% non-fat milk (for eNOS, gp91^phox^, 4-HNE, Akt, caveolin-1, and β-actin) in Tris-buffered saline or Odyssey blocking buffer (Cat. No. 927-50000; Li-Cor, Lincoln, NE; for adropin and peNOS^Ser1176^) before overnight incubation at 4°C with antibodies against either adropin (Cat. No. 14117, 1:1000; Cayman Chemical, Ann Arbor, MI), peNOS^Ser1176^ (Cat. No. 9571, 1:500; Cell Signaling Tech., Danvers, MA), eNOS (Cat. No. sc-654, 1:500; Santa Cruz Biotech., Dallas, TX), gp91^phox^ (Cat. No. 611414, 1:500; BD Biosciences, San Jose, CA), 4-HNE (Cat. No. ab48506, 1:200; Abcam, Cambridge, MA), Akt (Cat. No. 4691, 1:2000; Cell Signaling), caveolin-1 (Cat. No. ab2910, 1:2000; Abcam) or β-actin (Cat. No. A1978, 1:10000; Sigma-Aldrich, Saint Louis, MO). Afterwards, membranes were washed and incubated for 1 h with goat anti-rabbit IRDye 800CW (1:30000; Li-Cor, Lincoln, NE), goat anti-mouse IRDye 800CW (1:30000; Li-Cor) or donkey anti-mouse IRDye 680LT (1:40000; Li-Cor) secondary antibodies. Immunoreactive bands were visualized and densitometrically analyzed using Odyssey infrared scanner and Image Studio 2.0 software (Li-Cor).

### Measurement of adropin levels in rat plasma

Adropin levels in rat plasma were quantified using an ELISA kit (Cat. No. EK-032-35, Phoenix Pharmaceuticals, Inc., Burlingame, CA) as recommended by the manufacturer’s protocol. Twenty microliters of rat plasma were diluted with 80 µl of assay buffer and then added to the appropriate microtiter wells. All samples were assayed in triplicate and optical absorbance at 450 nm was measured with a Synergy™ HT Multi-Mode Plate Reader (Biotek Instruments, Winooski, VT). A standard curve was constructed (0.01-100 ng/ml) and the concentrations of adropin in plasma samples were determined from the standard curve.

### Statistical Analysis

All data were analyzed with GraphPad Prism 6 and expressed as mean ± SEM. Statistical analysis was performed by unpaired Student’s *t*-test and *P*<0.05 was considered statistically significant.

## RESULTS

### Aged-related decrease in Enho mRNA and adropin protein levels

**Figure 1.**
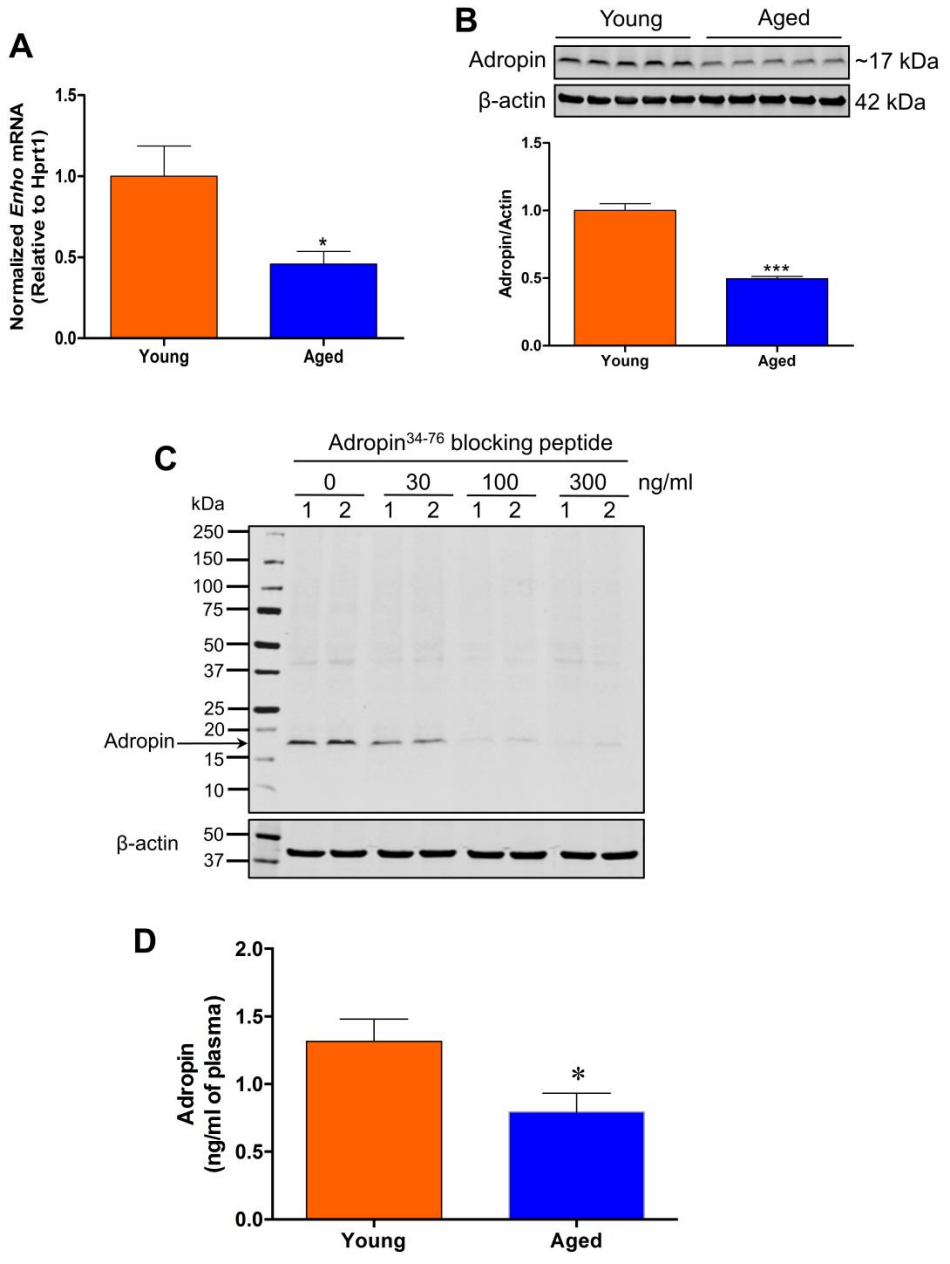
*Enho* mRNA and protein levels are downregulated in the aged rat brain and are associated with decreased adropin levels in plasma A) *Enho* mRNA expression was significantly decreased in the cerebral cortex of aged rats compared to young rats. B) Cortical homogenates (50 µg of total protein) from rat brains were subjected to SDS-polyacrylamide gel electrophoresis and endogenous adropin protein levels were detected by Western blot. Representative immunoblots and densitometric data showed that adropin protein levels in the cerebral cortex were dramatically decreased in aged rats compared to young rats. C) Adropin antibody is highly specific. Cortical brain homogenates (samples 1 and 2) were denatured at 70°C for 10 min in 2X Laemmli sample buffer containing 4% β-mercaptoethanol, separated on 4-20% SDS-polyacrylamide gels, and then transferred onto nitrocellulose membranes. Before incubation with the membrane, the adropin monoclonal antibody was pre-absorbed with different concentrations of synthetic adropin^34-76^ peptide (0-300 ng/ml), which resulted in a concentration-dependent loss of the intensity of the band corresponding to endogenous brain adropin. D) Plasma adropin levels were measured by a commercial adropin ELISA kit. A significant decrease in adropin levels was observed in aged rats compared with young controls. *P<0.05, ***P<0.001 vs. young rats. n=5 for young rats, and n=5 for aged rats.

We quantified *Enho* gene and protein expression in the brain and measured plasma adropin levels in young and aged naïve rats. *Enho* was dramatically downregulated in the cerebral cortex of aged rats (Fig. 1A), which was associated with decreased adropin protein levels measured by Western blot when compared to the young animals (Fig. 1B). We confirmed the specificity of the monoclonal antibody used to detect adropin in the brain lysate. Pre-absorption of the antibody with synthetic adropin^34-76^ peptide (Cat. No. 032-35, Phoenix Pharmaceuticals Inc., Burlingame, CA) resulted in a concentration-dependent loss of the signal corresponding to the endogenous brain adropin (Fig. 1C), which strongly indicates that the adropin monoclonal antibody used in our study is highly specific. Additionally, we found significantly reduced adropin levels in the plasma of aged rats compared to young animals (Fig. 1D).

### Markers of endothelial dysfunction and oxidative stress are increased in the aged rat brain

**Figure 2.**
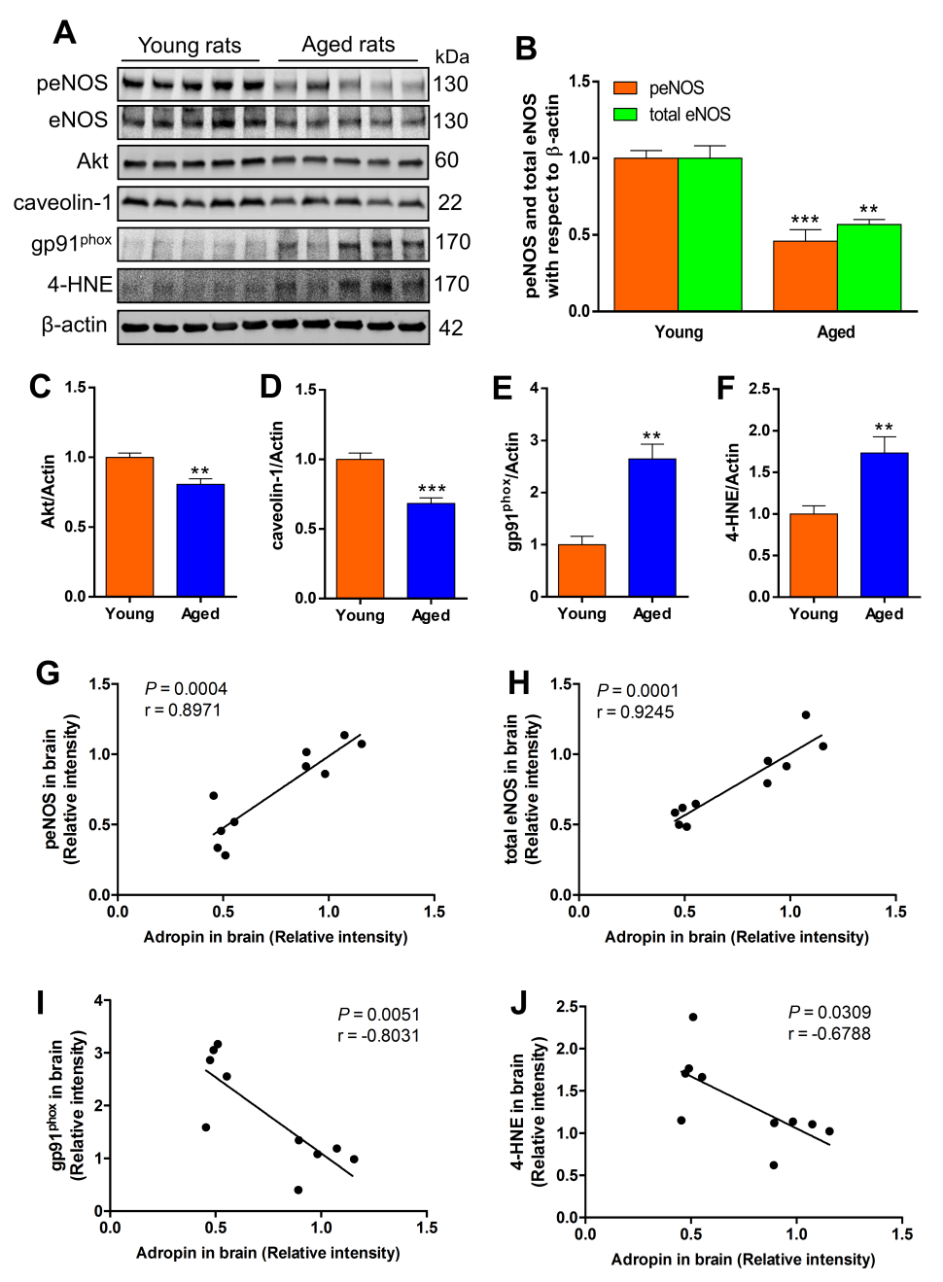
Biological markers of endothelial dysfunction and oxidative stress are increased in the aged rat brain Rat brain cortical homogenates (50 µg of total protein) were subjected to SDS-polyacrylamide gels and several biological markers for endothelial function and oxidative stress were detected by western blot. (A) Representative immunoblots for phosphorylated eNOS, total eNOS, Akt, caveolin-1, gp91^phox^ catalytic subunit of NADPH oxidase, and 4-hydroxy-2-nonenal (4-HNE)-modified proteins in the cerebral cortex of young and aged rats. Densitometric analysis showed that total and phosphorylated levels of eNOS (B), as well as levels of Akt (C) and caveolin-1 (D) were significantly decreased, while oxidative stress markers including gp91^phox^ (E) and 4-HNE-modified proteins (F) were significantly increased in aged rat brains compared to young rats. **P<0.01, ***P<0.001 vs. young rats. n=5 for young rats, and n=5 for aged rats. Positive correlation between brain adropin levels and phospho-eNOS (G) and total eNOS (H). A statistically significant negative correlation was found between brain adropin levels and gp91^phox^ (I) and 4-HNE levels (J). Pearson correlation coefficient (r) and p values are indicated for each correlation analysis.

Because our previous experiments demonstrated that adropin-mediated neuroprotection in the ischemic mouse brain is through eNOS activation and reduced oxidative damage [9], we asked whether reduced adropin in the aged rat brain is associated with changes in eNOS and oxidative stress. As shown in Fig. 2A and 2B, we found that phospho- and total-eNOS in the cerebral cortex were dramatically decreased in aged rats compared to young rats. Since Akt and caveolin-1 have been shown to modulate eNOS function [15], we next asked whether age alters these two proteins in the brain. We found that both Akt (Fig. 2A and 2C) and caveolin-1 (Fig. 2A and 2D) protein levels were significantly reduced in the aged rat brains compared to young rats, suggesting that aging is associated with significant alterations in these two eNOS regulatory proteins.

To determine oxidative damage, we first measured the levels of gp91^phox^, the catalytic subunit of NADPH oxidase that is a major source of superoxide radical generation [16], and found that it was significantly increased in aged compared to young rat brains (Fig. 2A and 2E). Furthermore, we investigated the effect of aging on lipid peroxidation, as assessed by quantification of 4-hydroxynonenal (4-HNE), a major cytotoxic lipid peroxidation product from omega 6-polyunsaturated fatty acids [17]. As shown in Fig. 2A and 2F, we found a significant increase in 4-HNE in the cerebral cortex of aged rats compared to young animals.

We next performed correlation analyses between adropin levels in the brain and peNOS, total eNOS, gp91^phox^ and 4-HNE levels. We found a statistically significant positive correlation between brain adropin levels and peNOS (Fig. 2G) as well as for total eNOS (Fig. 2H). A statistically significant negative correlation was found between brain adropin levels and measures of oxidative stress indicating that age-dependent decrease in brain adropin is associated with increased gp91^phox^ (Fig. 2I) and increased lipid peroxidation as assessed by 4-HNE levels (Fig. 2J).

## DISCUSSION

In the present study, we compared *Enho* mRNA and protein levels in brain as well as plasma adropin in young and aged rats. We found that both *Enho* mRNA and protein are significantly downregulated in aged rat brains compared to those of young animals in addition to reduced plasma adropin in aged rats. Additionally, total and phosphorylated eNOS were significantly decreased in aged rat brains, and this was associated with dramatic increases in oxidative damage markers, gp91^phox^ and 4-HNE, which are important contributors to endothelial dysfunction.

Adropin is abundantly expressed in the central nervous system (CNS), including many areas of the midbrain and hindbrain [2, 18]. Since its discovery in 2008, adropin has been shown to improve peripheral endothelial dysfunction, reduce insulin resistance, and increase glucose utilization in obesity and diabetes [1, 3, 19, 20], and clinical studies have found a significant association between altered plasma adropin and the risk of ischemic heart diseases [5, 8, 21, 22]. However, little is known about the effects of this functional peptide in the brain and what is the potential receptor for it. Wong et al. reported that adropin was a membrane-anchored protein highly expressed in the brain, which strongly binds to NB-3 (a brain-specific membrane-bound protein) to regulate Notch1 signaling in brain development, thus contributing to the locomotor activity and coordination of animals [2]. As such, impaired locomotor activity and coordination were observed in adropin deficient mice, which may be due to defects in the NB-3-mediated Notch1 signaling pathway [2]. Recently, Stein et al. demonstrated a central action of adropin in rat brain, where intracerebroventricular injection of adropin peptide into the lateral ventricle caused the inhibition of water intake and siRNA knockdown of GPR19 (an orphan G protein-coupled receptor), a candidate receptor for adropin, attenuated the inhibitory effects adropin on water deprivation-induced thirst [23]. A very recent study revealed that adropin in rat brain may exert its physiological function through direct effects on the excitability of hypothalamic paraventricular nucleus neurons [24]. Although these findings have demonstrated the possible physiological actions of brain adropin, it is not known whether the levels of adropin in the brain are changed under pathophysiological conditions. Our recent findings have demonstrated that *Enho* mRNA and protein levels are downregulated in the ischemic mouse brain, and treatment with synthetic adropin peptide can significantly reduce stroke-induced infarct volume, which was associated, in part, with restoration of endogenous adropin levels and increase in eNOS phosphorylation in the brain [9]. Since age is the single most important risk factor in ischemic stroke [25], it is of great clinical significance to know if age changes brain levels of adropin. A recent study has demonstrated that plasma adropin levels were decreased with age in humans and were associated with metabolic disorders risk factors such as dyslipidemia and insulin resistance [10]. Consistent with these findings, we found for the first time reduced plasma adropin in aged rats when compared to the young animals. Additionally, both *Enho* mRNA and protein were significantly downregulated in aged rat brains.

It is well-known that vascular endothelial dysfunction and oxidative stress occur during the human aging process [26, 27], and these can potentially contribute to age-related neuropathology. There is increasing evidence indicating that impaired NO production from eNOS is a central mechanism of endothelial dysfunction caused by aging [15, 28-32]. In eNOS^+/-^ mice, spontaneous thrombotic cerebral infarctions increased with age and these were associated with progressive cerebral amyloid angiopathy, blood-brain barrier breakdown, and cognitive impairment [33]. Increased eNOS activity is protective in the context of brain ischemia [34, 35] and eNOS deficient (eNOS^-/-^) mice have a worse stroke outcome [36]. Also, eNOS^-/-^ mice had increased amyloid precursor protein (APP), β-site APP-cleaving enzyme 1 (BACE1), and β-amyloid peptide (Aβ) in their brains compared to those of wild-type mice [37, 38], which contribute to the pathogenesis of Alzheimer’s disease (AD). Collectively, these findings strongly suggest that maintaining eNOS-dependent cerebrovascular endothelial function is of great importance in the prevention of stroke and AD.

However, the effects of aging on eNOS expression and activation in the brain remain to be elucidated. Strosznajder et al. reported that the brains of aged rats (24-month-old) had increased eNOS mRNA in the cerebral cortex, hippocampus and cerebellum compared to 4-month-old rats. Although there was no significant difference between eNOS protein levels in the aged and young brains, the aged cerebellum has significantly decreased eNOS activity compared to its younger counterpart [39]. Age-related eNOS differences in the brain may be specific to particular brain structures/regions because there was no significant difference in eNOS protein in prefrontal cortex of aged (24-month-old) rats versus young (4-month-old) animals [40]. Interestingly, we found that total and phosphorylated levels of eNOS in the cerebral cortex were significantly lower in aged rats than in young controls, which is consistent with a previous report showing that aging can significantly decrease eNOS protein levels in aged (23-24 months) rat brain microvessels as compared with young (4-6 months) animals [41]. We also found that gp91^phox^ (a major source of superoxide generation) and the lipid peroxidation product, 4-HNE, were dramatically increased in the cerebral cortex of aged rats compared to young animals, and these both contribute to endothelial dysfunction since oxidative damage induced by gp91^phox^ or 4-HNE can impair eNOS function by oxidizing tetrahydrobiopterin (BH4), an essential co-factor for this enzyme’s activity [42, 43]. Besides the detrimental effects on endothelial function, gp91^phox^ and 4-HNE contribute to the pathogenesis and progression of stroke and AD [44-47].

We found that aging was associated with reduced levels of Akt and caveolin-1, which have been shown to modulate eNOS activation [15]. Age-related alterations in Akt signaling have been previously reported in the brain [48]. Similarly, loss of caveolin-1 occurs in aged mice [49]. Loss of brain adropin levels, together with decreases in Akt, caveolin-1, and eNOS expression in the aged rat brain suggest the possibility that adropin might be involved in Akt/eNOS signaling pathway, as reported before in peripheral endothelial cells *in vitro* [3], or that adropin might alter the interaction between caveolin-1 and eNOS, thus modulating eNOS activity.

While we demonstrate for the first time that aging is associated with decreased *Enho* mRNA and protein levels in the rat brain as well as reduced adropin levels in plasma, there are some limitations in the current study. First, the loss of adropin in specific cell types was not identified in our study, which would have provided information of potential cellular and physiological changes related to reduced adropin in the aged brain. Second, our study only investigated the correlations between adropin levels and total/phospho-eNOS as well as oxidative stress markers, gp91^phox^ and 4-HNE, in young and aged rat brain. Due to the complexity of cellular and metabolic changes during the aging process it is difficult to demonstrate causality in our study. It remains to be investigated whether the loss of adropin results in reduced eNOS expression/phosphorylation, increased oxidative stress, and impaired endothelial function associated with aging. Since the identity of the adropin receptor remains elusive, the molecular mechanisms underlying its biological effects are currently poorly understood. We have previously found that eNOS is required for the adropin-mediated neuroprotection in stroke [9]. Future studies are warranted to identify the causal link between adropin levels and eNOS activation in the young and aged brain.

In conclusion, we report for the first time that *Enho* mRNA and protein levels are downregulated in aged rat brains compared with young controls, and this is associated with decreased plasma adropin levels. Further, we found that total and phosphorylated eNOS were significantly decreased, while oxidative stress markers including gp91^phox^ and 4-HNE were dramatically increased in aged rat brains. The findings from this study suggest that age-mediated loss of adropin in brain and plasma is associated with impaired eNOS function and increased oxidative stress, which may contribute to cerebrovascular disease.
